# Programmed death‐ligand 1 expression in patients with primary or secondary myelofibrosis

**DOI:** 10.1002/cnr2.2054

**Published:** 2024-09-05

**Authors:** Moayed Ibrahim, Catherine Murphree, Kirtesh Patel, Matthew Mastrodomenico, Nakhle S. Saba, Hana Safah, Janet Schmid, Francisco Socola

**Affiliations:** ^1^ Section of Hematology and Medical Oncology, Deming Department of Medicine Tulane University New Orleans Louisiana USA; ^2^ Delta Pathology Group Shreveport Louisiana USA; ^3^ Pathology Department Tulane University New Orleans Louisiana USA; ^4^ Hematology and Bone Marrow Transplant University of California Davis Sacramento California USA

**Keywords:** immunotherapy, JAK/STAT pathway, PD‐L1, primary myelofibrosis, secondary myelofibrosis

## Abstract

**Background:**

It has been described in mice models that myeloproliferative neoplasm (MPN) with JAK2‐V617F mutation has an increased expression of programmed death‐ligand 1 (PD‐L1) in megakaryocytes leading to cancer immune evasion by inhibiting the T‐lymphocytes.

**Aims:**

To quantify and compare the PD‐L1 expression on bone marrow (BM) of patients with MPN JAK2 positive, negative, and normal controls.

**Methods:**

We collected BM of patients with MPN JAK2 positive, negative and normal controls from 1990 to 2019. We also created a scoring system to quantify PD‐L1 expression in megakaryocytes.

**Results:**

We obtained 14 BM with JAK2 positive PMF, 5 JAK2 negative PMF, and 10 patients with normal BM biopsies. PD‐L1 expression was higher in the JAK2 positive group compared with the control group with a score of 212.6 versus 121.1 (*t*‐value 2.05, *p*‐value 0.025). In addition, the score was higher in the PMF group regardless of JAK2 mutational status when compared with the control group with score of 205.9 versus 121.1 (*t*‐value 2.12, *p*‐value 0.021). There was no difference in the PD‐L1 score between the JAK2 negative versus the control group 187.2 versus 121.1 (*t*‐value 1.02, *p*‐value 0.162).

**Conclusion:**

These findings suggest that PMF patients with a JAK2 mutation have a higher PD‐L1 expression in megakaryocytes compared with the control group. We postulate that the combination of checkpoint and JAK2 inhibitors may be an active treatment option in JAK2 mutated PMF given the higher PD‐L1 expression.

## 1 INTRODUCTION

Primary myelofibrosis (PMF) is a myeloproliferative neoplasm (MPN) characterized by constitutional activation of the JAK–STAT signaling pathway and bone marrow (BM) fibrosis, which leads to decreased peripheral blood counts, proinflammatory state, and a potential for transformation to acute myeloid leukemia. The median age at presentation is 65 years.^1^, ^2^ JAK2‐V617F is the most common mutation in PMF and is found in 50%–60% of patients.^3^ The only curative treatment option currently available is allogeneic stem cell transplant. However, most patients are ineligible because of advanced age and comorbidities.^4^ Ruxolitinib and fedratinib, JAK2 inhibitors, are the only FDA approved treatment for intermediate/high risk PMF patients.^5^, ^6^ However, those drugs have their limitations and only improve symptoms and decrease splenomegaly, without an overall survival benefit.^5^, ^6^ Therefore, there is a significant unmet need for treatment options in this patient population.

Prestipino et al. discovered that mice models with JAK2‐V617F mutated MPN generally have an increased expression of programmed death‐ligand 1 (PD‐L1) that leads to cancer immune evasion by inhibiting the antitumor effect of the T lymphocytes against cancer cells.^7^ Checkpoint inhibitors are monoclonal antibodies that block the interaction between PD‐L1 and its receptor, allowing the immune system to fight cancer cells with an enhanced antitumor response. Pembrolizumab was the first checkpoint inhibitor approved by the FDA for patients with metastatic nonsmall‐cell lung cancer with >50% PD‐L1 expression in tumor cells by immunohistochemistry stain. In this subset of patients, pembrolizumab was more effective than systemic chemotherapy.^8^

In this paper, we compared the PD‐L1 expression among patients with JAK2‐mutated PMF versus JAK2‐unmutated PMF patients versus normal controls without no PMF or JAK2 mutation.

## 2 PATIENT AND METHODS

### 2.1 Study population

We collected BM biopsies of patients with PMF done at Tulane Medical Center from 1990 to 2019. All these patients had a known JAK2 status and a well‐preserved specimen for adequate PD‐L1 staining. We only used the initial BM biopsy obtained to diagnose patients with PMF. We collected 10 additional samples of patients that came to clinic with transient cytopenias from benign hematologic conditions. These patients had normal karyotype, FISH panel for myelodysplastic syndrome, and a negative 64‐gene next generation sequencing (NGS) myeloid mutation panel. An institutional review board approval was obtained before collecting these BM samples and all participants gave informed consent. The authors analyzed the data to which all authors had access. All relevant participant data was deidentified and shared as appropriate in the text.

### 2.2 PD‐L1 immunohistochemistry staining

We used the FDA approved test PD‐L1 IHC 28‐8 pharmDx to assess the PD‐L1 expression on the BM biopsies. This is a qualitative immunohistochemical assay, which uses monoclonal mouse anti‐PD‐L1 Clone 22C3 intended for use in the detection of PD‐L1 protein in formalin‐fixed, paraffin‐embedded tissues of different cancers such as nonsmall cell lung cancer and gastric/gastroesophageal junction adenocarcinoma. This test is used to identify patients who may be treated with pembrolizumab.

BM sections of 4–5 μm were made with tissues mounted on microscope slides then placed in a 58 ± 2°C oven for 1 h. The slides were stained with the PD‐L1 IHC 22C3 pharmDx reagent, and the samples were incubated. Finally, all the slides were numbered and labeled with codes.

### 2.3 Interpretation of PD‐L1 expression

The slides were reviewed and scored independently by two clinical pathologists specialized in interpreting PD‐L1 expression in solid tumors. PD‐L1 was scored according to the quantity and intensity in the megakaryocytic lineage of cells. The quantity of PD‐L1 expression was graded from 0 to 100% while the intensity was graded from 1+ to 3+, according to the level of intensity. The result was a PD‐L1 score calculated by multiplying percentage by intensity of PD‐L1 in the BM megakaryocytes. This score will be detailed further in the statistical analysis section.

### 2.4 JAK2 mutation and NGS myeloid mutation panel

Patients with PMF diagnosis had a JAK2‐V617F mutation status known while the patients diagnosed with PMF after 2018 also had a myeloid NGS done. The controls had the myeloid NGS done ensure they did not have any clonal diseases. The genes included in our in‐house myeloid NGS mutation panel are: ABL1, ASXL1, ATM, ATRX, BCOR, BCORL1, BRAF, CALR, CBL, CBLB, CDKN2A, CEBPA, CSF3R, DAXX, DNMT3A, EED, EGFR, ETV6, EZH2, FBXW7, FLT3, GATA1, GNAS, HRAS, IDH1, IDH2, IKZF1, JAK1, JAK2, JAK3, KAT6A, KIT, KMT2A, KRAS, MPL, NF1, NOTCH1, NPM1, NRAS, PDGFRA, PHF6, PRPF40B, PTEN, PTPN11, RAD21, RB1, RUNX1, SETBP1, SF1, SF3A1, SF3B1, SH2B3, SMARCB1, SMC1A, SMC3, SRSF2, STAG2, SUZ12, TET2, TP53, U2AF1, U2AF2, WT1, and ZRSR2.

## 3 RESULTS

**FIGURE 1:**
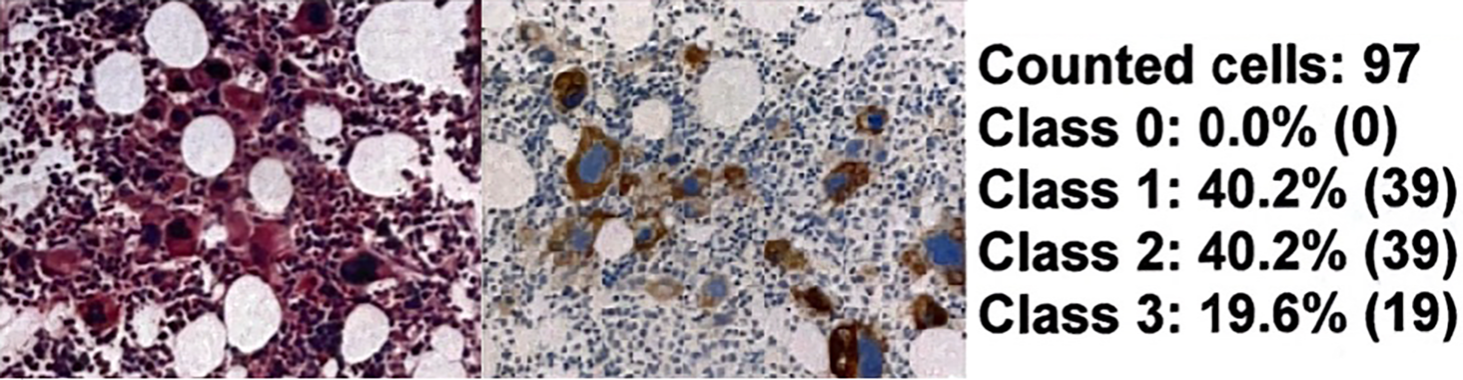
It represents the bone marrow BX of a 76‐year‐old woman with newly diagnose JAK2‐mutated PMF, and the megakaryocytes are hyperplastic with all of them expressing programmed death‐ligand 1 (PD‐L1) with the highest intensity. In this slide, the pathologist counted 97 cells. Expression was quantified as 97% (39 + 39 + 19), and intensity was very bright and for that reason, it was given the highest score of 3+. The PD‐L1 score was 291.

**FIGURE 2:**
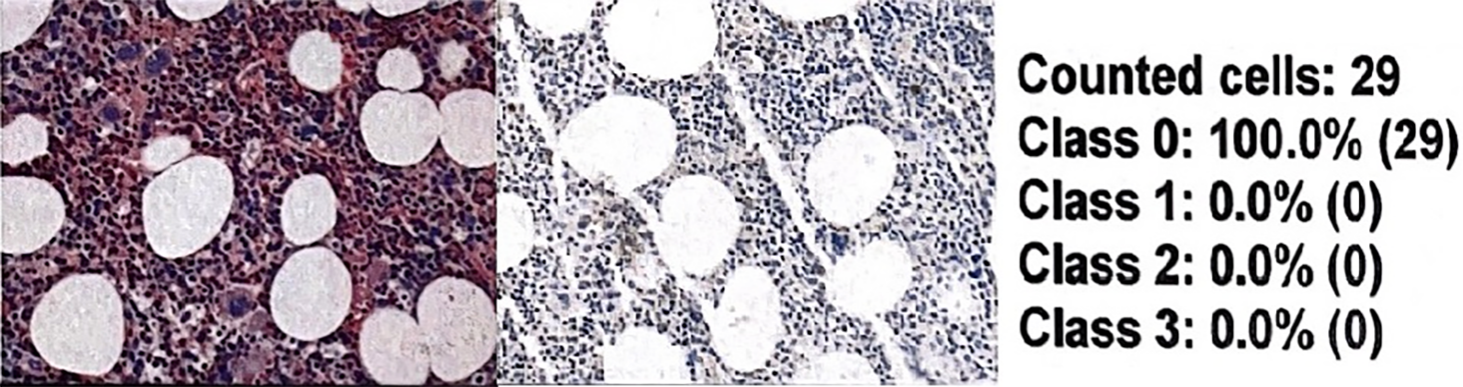
It represents a bone marrow BX of a 28‐year‐old woman with unexplained anemia in the slide the pathologist counted 29 megakaryocytes that looked normal and they did not highlight the programmed death‐ligand 1 (PD‐L1) stain, for that reason, the expression and intensity were score as 0. PD‐L1 score was 0.

**FIGURE 3:**
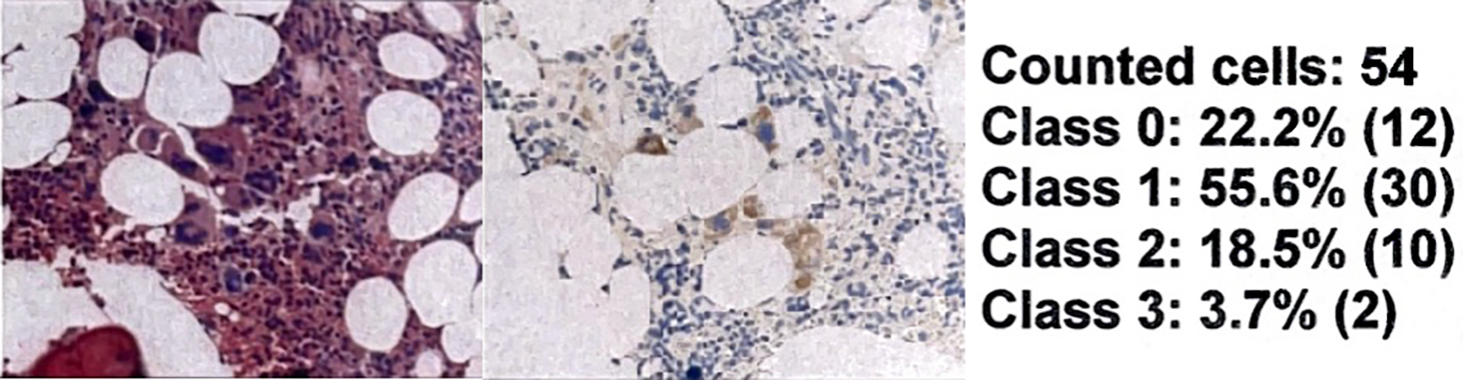
It represents a bone marrow BX of a 73‐year‐old woman with JAK2‐unmutated programmed death‐ligand 1 (PD‐L1) that reveals some hyperplastic megakaryocytes. In this slide, the pathologist counted 54 cells. Expression was quantified as 78% (55.6 + 18.5 + 3.7), and intensity was bright but not as intense as in the Figure 1, for that it was scored as 2+. The PD‐L1 score was 156.

**FIGURE 4:**
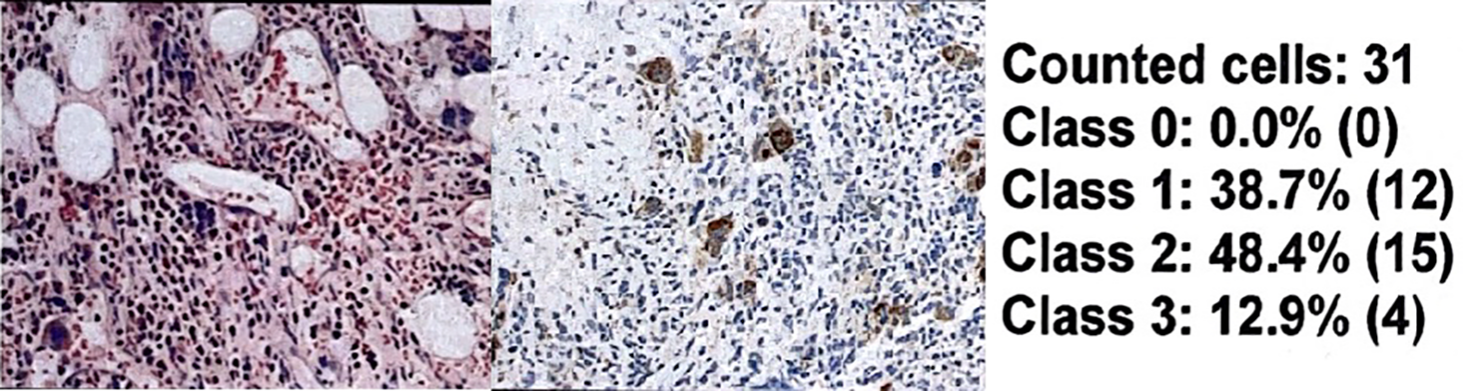
It represents a bone marrow BX of a 60‐year‐old man with JAK2‐unmutated primary myelofibrosis with hyperplastic megakaryocytes. In this slide, the pathologist counted 31 cells. Expression was quantified as 100% (38.7 + 48.4 + 12.9), and intensity was very bright, for that, it was scored as 3+. The programmed death‐ligand 1 score was 300.

We studied the BM biopsies of 29 patients in total; 14 patients had JAK2‐mutated PMF, 5 JAK2‐unmutated PMF, and 10 controls with negative BM biopsies. The median age of the whole group was 57 years, with 34% males and 66% females. The main clinical characteristics of the myelofibrosis patients are described in the Table 1. PD‐L1 expression and intensity are described in Table 2. We have a few examples how the PD‐L1 expression and intensity was quantified in the BM biopsies, as shown in Figures 1, 2, 3, 4.

**TABLE 1:** Main characteristics of patients with myelofibrosis.

Features	MPN
Age	57 years
Female	13 (66%)
Primary myelofibrosis (all of them overt PMF)	10 (52%)
Post‐PV MF	6 (32%)
Post‐ET MF	3 (16%)
Low risk	1 (5%)
Intermediate risk 1	7 (37%)
Intermediate risk 2	7 (37%)
High risk	4 (21%)

Abbreviations: MPN, myeloproliferative neoplasm; PMF, primary myelofibrosis; Post‐ET MF, post‐essential thrombocythemia myelofibrosis; Post‐PV MF, post‐polycythemia vera myelofibrosis.

**TABLE 2:** Level of expression and intensity of PD‐L1 on the 29 bone marrow biopsies.

#	Diagnosis	JAK2 status	PD‐L1 quantity %	Intensity+	Score = PD‐L1 × intensity
1	PMF	Negative	100	1	100
2	PMF	Negative	78	2	156
3	PMF	Negative	100	3	300
4	PMF	Negative	73	2	146
5	PMF	Negative	78	3	234
6	Control	NA	100	3	300
7	Control	NA	83	2	166
8	Control	NA	0	0	0
9	Control	NA	100	3	300
10	Control	NA	0	0	0
11	Control	NA	90	1	90
12	Control	NA	96	3	288
13	Control	NA	0	0	0
14	Control	NA	67	1	67
15	Control	NA	0	0	0
16	PMF	Positive	100	3	300
17	PMF	Positive	93	2	186
18	PMF	Positive	0	0	0
19	PMF	Positive	83	3	249
20	PMF	Positive	97	3	291
21	PMF	Positive	84	3	252
22	PMF	Positive	85	2	170
23	PMF	Positive	93	3	279
24	PMF	Positive	87	3	261
25	PMF	Positive	87	2	174
26	PMF	Positive	85	3	255
27	PMF	Positive	73	3	219
28	PMF	Positive	73	1	73
29	PMF	Positive	89	3	267

Abbreviations: PD‐L1, programmed death‐ligand 1; PMF, primary myelofibrosis.

The average PD‐L1 expression score for the JAK2‐mutated PMF group was 212.57, the JAK2‐unmutated PMF group 187.2, and the control group 121.1. There was a statistically significant difference between the PD‐L1 score between the JAK2‐mutated PMF group versus the control group (*t*‐value 2.05 and *p*‐value of .025) and when we compared the PMF group regardless of the JAK2 status versus the control group (*t*‐value 2.12 and *p*‐value of .021). However, there was not a statistically significant difference when we compared the PD‐L1 expression between the JAK2 negative versus the control group (*t*‐value 1.02 and *p*‐value .162).

**FIGURE 5:**
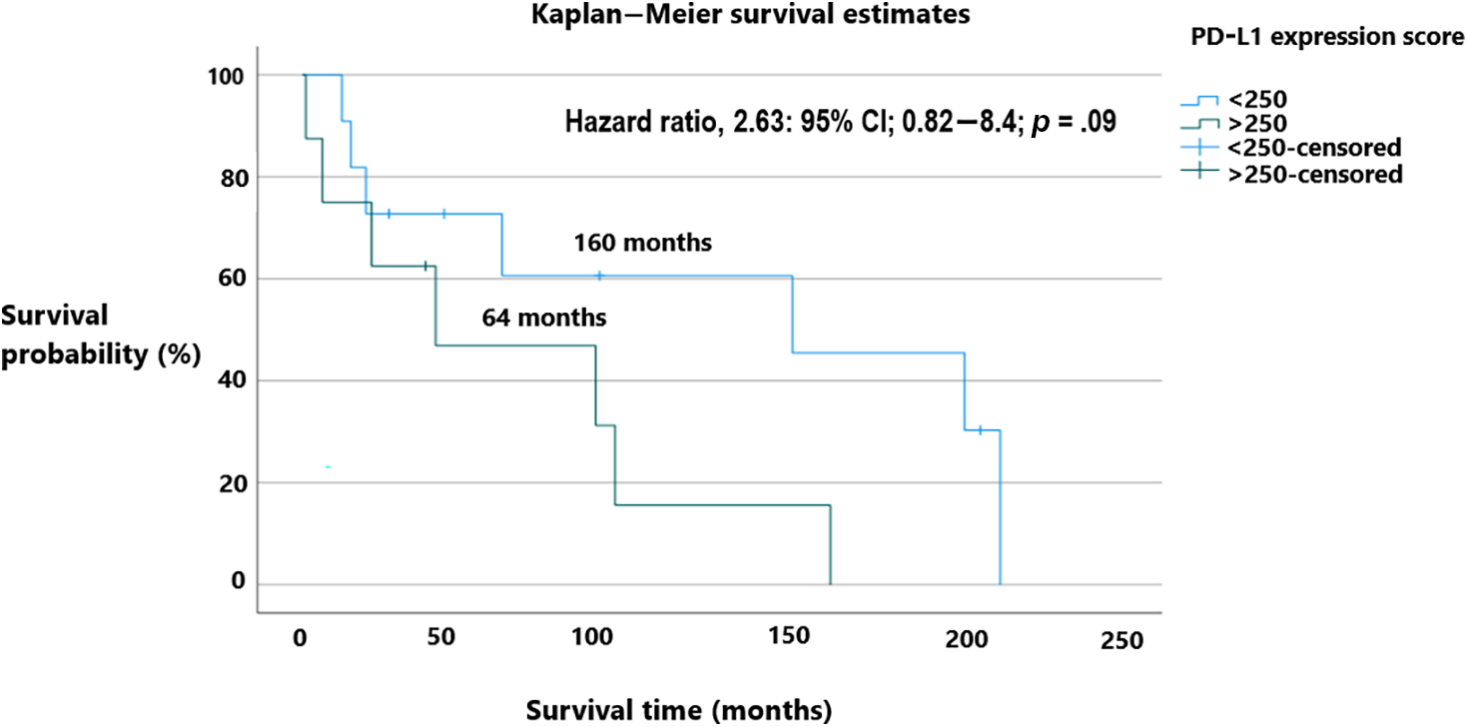
Median overall survival between programmed death‐ligand 1 (PD‐L1) expression score <250 (130 months) versus >250 (64 months).

Myelofibrosis patients with a PD‐L1 score <250 had a median overall survival of 130 months versus PD‐L1 score >250 of 64 months (hazard ratio 2.63, 95% CI; 0.82–8.4; *p* = .09), it was numerically longer but not statistically significant as shown in Figure 5.

## 4 DISCUSSION

The presented results show that PD‐L1 score was higher in the PMF group versus the control group regardless of JAK2 mutation status, which turned out to be statistically significant. This helps build on the data reported by Prestipino et al. that showed oncogenic JAK2 activity led to STAT phosphorylation, which in turn enhanced PD‐L1 promoter activity and PD‐L1 protein expression in JAK2 mutant cells.^7^ In addition, PD‐L1 expression was higher on primary cells isolated from patients with JAK2‐mutated MPNs as compared with healthy individuals and declines upon JAK2 inhibition.^7^ Moreover, Lee et al. were able to demonstrate that PD‐L1 expression was significantly associated with overt myelofibrosis and JAK2 mutational status.^9^ Moreover, in the previously mentioned study, there were four patients who were found to have a particularly high PD‐L1 expression that also harbored the JAK2 mutation.^9^

This supports further that PD‐L1 may play a more important role than previously realized in MPNs and should perhaps be a future target in our current small armamentarium of viable drugs. To the best of our knowledge, there have only been two small phase II trials, in which the investigators tested the utility of PD‐L1 inhibition (pembrolizumab and nivolumab) in patients with PMF. Hobbs et al. conducted a phase 2, single arm study of pembrolizumab in patients with Dynamic International Prognostic Scoring System (DIPSS) intermediate‐2 or greater, primary, or secondary, post essential thrombocythemia or post polycythemia vera MF who were ineligible for or previously treated with ruxolitinib.^9^ This study had 10 patients, 5 with JAK2 mutation who were treated with pembrolizumab without objective clinical responses. However, an important takeaway from this data showed that flow cytometry, T‐cell receptor sequence, and proteomics demonstrated changes in the immune makeup of patients, suggesting improved T cell responses.^10^ Although this study was terminated early as no objective clinical responses were seen, the latter changes mentioned suggest that perhaps PD‐L1 inhibition is not enough to elicit a clinical response and combination therapy may be more effective.

Another study, in which Dall et al. investigated the efficacy and safety of single agent nivolumab in eight adult patients with myelofibrosis, was also terminated early due to failure to meet predetermined efficacy endpoint (primary endpoint was objective response rate defined as complete response, partial response, and clinical improvement after eight doses).^11^ The median duration of enrolled patients on the study was 5.4 months with a median number of cycles of 3. Unfortunately, in this study, none of the patients responded to nivolumab therapy. These patients showed more advanced disease including intermediate two and high risk DIPSS score with five patients failing ruxolitinib and seven with clonal evolution, that is, progressive disease.^11^

It is important to note that these two described studies had very small sample sizes, with most patients in the high risk DIPSS category, multiple previous lines of therapy, and complex mutational status or clonal evolution. However, they were able to characterize changes in the patient's immune milieu after administration of PD‐L1 blockade.^10^, ^11^ It is noteworthy to the authors that both studies employed PD‐L1 blocked only after patients had undergone multiple lines of therapy, in a relapsed or refractory setting, raising the question that perhaps this is not the right setting to use this line of therapy. In addition, it would be interesting to expand further on the hypothesis that perhaps PD‐L1 blockade is not enough and combination therapy with ruxolitinib may be more effective in patients with PMF.

## 5 CONCLUSIONS

We found that the PD‐L1 expression in the BM megakaryocytes of JAK2‐mutated PMF patients was higher than the control group. This may confirm the hypothesis that the oncogenic JAK2 mutation enhances the PD‐L1 promoter activity and its expression in JAK2 positive patients. We hypothesize that patients with high PD‐L1 expression may benefit from checkpoint inhibitors, in combination with JAK2 inhibitors, in the upfront setting of therapy.

## AUTHOR CONTRIBUTIONS

**Moayed Ibrahim:** Conceptualization (equal); data curation (equal); formal analysis (equal); investigation (equal); methodology (equal); project administration (equal). **Catherine Murphree:** Conceptualization (equal); data curation (equal); formal analysis (equal); investigation (equal); methodology (equal); project administration (equal). **Kirtesh Patel:** Conceptualization (equal); data curation (equal); formal analysis (equal); investigation (equal); methodology (equal); project administration (equal). **Matthew Mastrodomenico:** Conceptualization (equal); data curation (equal); formal analysis (equal); investigation (equal); methodology (equal); project administration (equal). **Nakhle S. Saba:** Conceptualization (equal); data curation (equal); formal analysis (equal); funding acquisition (equal); investigation (equal); methodology (equal); project administration (equal). **Hana Safah:** Conceptualization (equal); data curation (equal); formal analysis (equal); funding acquisition (equal); investigation (equal); methodology (equal); project administration (equal). **Janet Schmid:** Conceptualization (equal); data curation (equal); formal analysis (equal); funding acquisition (equal); investigation (equal); methodology (equal); project administration (equal). **Francisco Socola:** Conceptualization (lead); data curation (lead); formal analysis (lead); funding acquisition (lead); investigation (lead); methodology (lead); project administration (lead).

## CONFLICT OF INTEREST STATEMENT

The authors have stated explicitly that there are no conflicts of interest in connection with this article.

## ETHICS STATEMENT

The study was conducted in accordance with the Declaration of Helsinki, and the protocol was approved by the Ethics Committee of Tulane University.

## Data Availability

Data sharing is not applicable to this article as no new data were created or analyzed in this study.
